# Unlocking Dendritic Cell-Based Vaccine Efficacy through Genetic Modulation—How Soon Is Now?

**DOI:** 10.3390/genes14122118

**Published:** 2023-11-23

**Authors:** Ahmed Elwakeel, Hannah E. Bridgewater, Jason Bennett

**Affiliations:** 1 Centre for Health and Life Sciences (CHLS), Coventry University, Coventry CV1 5FB, UK; elwakeela@uni.coventry.ac.uk (A.E.); ad4698@coventry.ac.uk (H.E.B.); 2 Department of Biological Sciences, Faculty of Science and Engineering, University of Limerick, V94 T9PX Limerick, Ireland

**Keywords:** dendritic cells, dendritic cell-based vaccines, genetic engineering, RNAi, CRISPR gene editing

## Abstract

The dendritic cell (DC) vaccine anti-cancer strategy involves tumour-associated antigen loading and maturation of autologous ex vivo cultured DCs, followed by infusion into the cancer patient. This strategy stemmed from the idea that to induce a robust anti-tumour immune response, it was necessary to bypass the fundamental immunosuppressive mechanisms of the tumour microenvironment that dampen down endogenous innate immune cell activation and enable tumours to evade immune attack. Even though the feasibility and safety of DC vaccines have long been confirmed, clinical response rates remain disappointing. Hence, the full potential of DC vaccines has yet to be reached. Whether this cellular-based vaccination approach will fully realise its position in the immunotherapy arsenal is yet to be determined. Attempts to increase DC vaccine immunogenicity will depend on increasing our understanding of DC biology and the signalling pathways involved in antigen uptake, maturation, migration, and T lymphocyte priming to identify amenable molecular targets to improve DC vaccine performance. This review evaluates various genetic engineering strategies that have been employed to optimise and boost the efficacy of DC vaccines.

## 1. Introduction

In 1973, when Ralph Steinman focused his microscope on a distinct cell type in a heterogeneous population of mouse splenocytes, even the most optimistic scientific mind could not envisage the impact this discovery would have on the field of immunology. These unusual cells possessed a distinctive tree-like morphology, motile capacity, and extensive mitochondria. Due to their distinct morphology, Steinman and Zanvil Cohn named these cells dendritic cells (DCs) after the Greek word “dendron” meaning tree [1]. Subsequent early experiments confirmed DCs as the most potent inducers of the mixed lymphocyte reaction and key immune response initiators, with the ability to initiate adaptive immune responses and induce allogeneic T cell responses [2]. Five decades of research have proven DCs to be highly specialised antigen-presenting cells (APCs) that bridge the gap between the innate and adaptive immune systems. Due to their efficient phagocytic activity, DCs act as “sentinels” of the immune system, sampling antigens in the periphery in order to seek out pathogenic cues in tissue microenvironments and inform the adaptive immune system [3]. Developmentally, DCs can be initially classified into immature and mature cells. Immature DCs are widely distributed at sites of potential antigen exposure and have full antigen-endocytic capabilities with low expression levels of major histocompatibility complex (MHC) and co-stimulatory molecules [4]. They also have a limited proinflammatory cytokine production capacity. Maturation is triggered upon antigen capture and recognition of danger-associated molecular patterns (DAMPs) and pathogen-associated molecular patterns (PAMPs) [4]. DC maturation commences with the processing of engulfed antigen into peptides and their subsequent presentation, linked to MHC molecules, on the cell surface. Crucially, maturation signals induce the expression of co-stimulatory molecules (CD80, CD86, and CD40), chemokine receptors (e.g., CCR7), pro-inflammatory cytokines, and ultimately migration from peripheral tissues via lymphatic vessels into draining lymph nodes, where they activate tissue-specific T cell responses [4]. T cell activation is tightly regulated and requires the presence of three sequential signals—“three-signal theory”. T cell activation depends on antigen presentation (signal 1), binding of co-stimulatory molecules (signal 2), and proinflammatory cytokine secretion (signal 3). Immune tolerance occurs when there is antigen presentation in the absence of signals 2 and 3 [2].

With the exponential expansion in DC biology research, it became evident that “DCs” is an umbrella term to describe a heterogeneous family of cells derived from hematopoietic progenitors that produce multiple distinct functional subsets capable of generating diverse immunological responses. An initial classification scheme based on source, phenotype, and function grouped human DCs into three main subsets: monocyte-derived DCs (MoDCs), conventional (or classical) DCs (cDCs), and plasmacytoid DCs (pDCs) [5]. However, issues arose in this general phenotypic-based and function-based classification system since environmental signals can influence and change DC functional roles and phenotypic markers. Independent of functional or phenotypic properties, DC subset identification is now based around ontogeny with distinct developmental precursors and specific transcription factors acting as lineage-determining factors through the induction of specific transcription programs. Six clusters of distinct human DC subtypes have emerged. MoDCs are a heterogeneous population of APCs that differentiate from monocytes in response to inflammation [6,7]. Conventional DCs CD141^+^ cDC1 are central to type-1 immunity, while CD1c^+^ cDC2 (which are further subdivided into cDC2A and CDC2B subtypes) specialise in priming Th17 and Th2 cell immune responses via antigen presentation on MHCII [8]. Plasmacytoid DCs play a major role in immune responses to viral infections due to their substantial IFN-I-producing ability. Finally, the newly discovered Axl + DCs, which are inefficient at IFN-I production but can activate T and B cell responses [9,10]. DC ontogeny and subtype complexity is an ever-evolving field that is not within the scope of this review but has been well characterised elsewhere [5,11,12]. This review endeavours to highlight various genetic engineering strategies that have been developed to boost the clinical efficacy of DC vaccines. However, firstly, it is important to provide an overview of the dysfunctional role of DCs in the tumour microenvironment (TME) and how the DC vaccine approach was developed to bypass the TME and drive anti-tumour T-cell responses.

## 2. Dendritic Cells in Cancer—A Tumour Microenvironment (TME)-Induced Dysfunctional Role

Cancer immunosurveillance—a concept originally postulated by Burnet and Thomas in the mid-1950s—describes how under physiological conditions our immune system can mount an anti-tumour response, specifically detecting and eliminating nascent tumour cells, thereby preventing tumour growth and inducing immunological memory to control future outgrowths [13]. Central to this process is the activation of tumour-specific CD8^+^ cytotoxic T lymphocytes following the presentation of tumour-associated antigens (TAAs) by APCs to naive T cells in draining lymph nodes. This is the fundamental initiation step in the cancer-immunity cycle [14]. Experimental confirmation of cancer immunosurveillance opened a door to harnessing the power of the immune system to eliminate tumours [15]. Cancer immunotherapy is now established as the 5th pillar of cancer care, joining the ranks of surgery, chemotherapy, radiation, and targeted therapies [16]. The development of effective immune checkpoint therapies (ICTs) is revolutionising the clinical management of certain malignancies, such as advanced metastatic melanoma and non-small cell lung cancer [17]. However, numerous challenges remain. Most patients undergoing ICT rarely exhibit an objective response due to the overriding immunosuppression mechanisms in the tumour microenvironment (TME) and the subsequent absence of T-cells from the TME [18,19,20]. Hence, additional adjunct strategies to boost anti-tumour immune responses are required to fully unlock the power of current immunotherapies in the clinic.

The central role of DCs in the cancer-immunity cycle has been confirmed in various model systems [21], and live imaging studies have confirmed DCs ability to uptake and process tumour antigens before migration to draining lymph nodes or tumour-associated tertiary lymphoid structures (TLS) for presentation to T cells [22,23]. However, immunosuppressive mechanisms of an established TME—a niche created by the confluence of tumour cells, supporting stroma, and infiltrating immune cells—induce DC dysfunction and represent a fundamental barrier to the efficacy of current DC-based therapies [24]. Multiple TME-generated cellular and soluble factors contribute to suppressing DC recruitment, activation, and antigen presentation. Immunosuppressive growth factors and cytokines (e.g., vascular endothelial growth factor (VEGF), macrophage colony-stimulating factor (M-CSF), interleukin-10 (IL-10), and transforming growth factor β (TGF-β)) are released by a heterogenous population of cells within the TME and exert an immunosuppressive influence by inhibiting DC differentiation and maturation [24]. Regulatory T cells (Tregs) represent a specialized subpopulation of T cells that maintain homeostasis and tolerance through immune response suppression and are key drivers of DC dysfunction in the TME [25,26,27]. Following recruitment and infiltration of Tregs into the TME through CCR4 chemokine signalling, Tregs proliferate under the influence of IL-10 and TGF-β [25,26,27]. Tregs’ ability to suppress pro-inflammatory cells in the TME (including DCs) is based on the further secretion of inhibitory cytokines or through direct cell-to-cell contact via inhibitory receptors such as programmed cell death 1 (PD-1) and cytotoxic T-lymphocyte-associated protein 4 (CTLA-4) [28]. Additionally, alterations of tumour antigens in the TME allow tumour cells to evade DC detection. Indeed, Jaeger and colleagues (2019) recently highlighted a unique role for the molecular chaperone HSP90 in “hiding” tumour antigens from the antigen presentation pathway by stabilising them [29]. Additional examples of antigen masking include the inability of DCs to process and present post-translationally hypoglycosylated MUC1 (hgMUC1) to T cells [30] and the ability of the hepatocellular carcinoma (HCC)-derived fucosylated variant of the oncofoetal tumour antigen, α-fetoprotein (AFP), to reduce DC maturation marker levels, pro-inflammatory mediator expression, and dampen T cell proliferative responses [31]. Further studies by Munson et al. (2023) demonstrated that phagocytosis of HCC-derived AFP altered DC metabolism, decreased expression of co-stimulatory molecules, and increased expression of immune checkpoint molecules such as PD-L1 [32]. Other TME-related characteristics reported to inhibit DC functions directly or indirectly include hypoxia, metabolic stress, and lipid accumulation within DCs [24,33].

The TME-based immunosuppressive mechanisms represent a significant barrier to normal DC function. However, due to their superior capacity to acquire, process, and present TAAs to effector T cells, DCs remain a potential key weapon in the cancer immunotherapy arsenal and have been the focus of translational efforts designed to boost effector T cell responses to tumours. Various strategies have been employed to modulate the activity of tumour-infiltrating dendritic cells in vivo. These strategies include administration of Toll-like receptor (TLR) ligands, intratumoural injection of TriMix mRNA (mRNA encoding the co-stimulatory molecule CD70, the activation stimuli CD40 ligand, and constitutively active Toll-like receptor 4) and attenuated viral agents [24,34,35]. Specifically, TriMix administration was demonstrated to re-programme intratumoural DCs into mature APCs capable of migrating to draining lymph nodes and initiating anti-tumour T cell responses [34]. Furthermore, administration of the FMS-like tyrosine kinase 3 ligand (FLT3L) was demonstrated to increase cDC proliferation and ultimately tumour infiltration in a mouse model of melanoma [36]. Additional research and positive clinical trial results will be required to confirm the feasibility of these endogenous tumour-infiltrating DC targeting strategies. Regardless, in order to circumvent the immunosuppressive influence of the TME on endogenous DC maturation and activation status, the DC vaccination approach was developed.

## 3. DC-Based Anticancer Vaccines “101”

**Figure 1:**
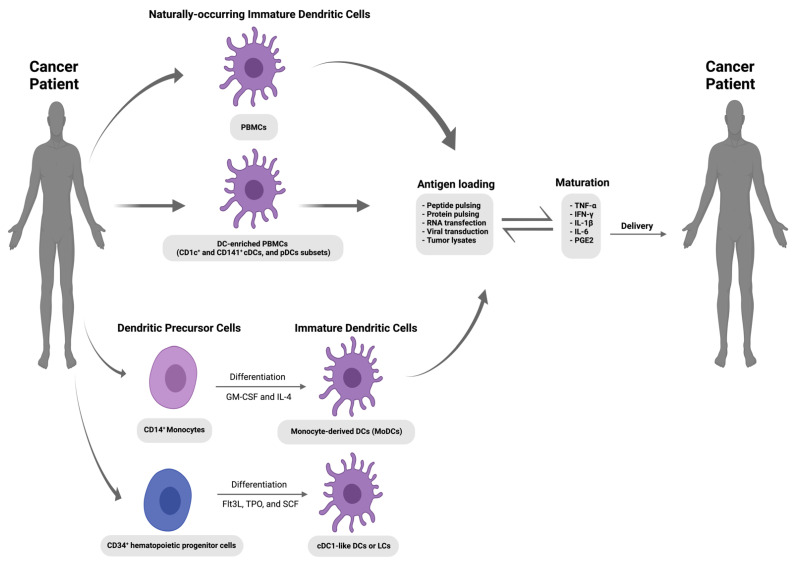
Schematic representation of the DC-based anti-cancer vaccination strategy. Immature dendritic cells are isolated from blood (peripheral blood mononuclear cells [PBMCs] or DC-enriched PBMCs) or derived from blood monocytes (MoDCs) and CD34^+^ hematopoietic stem progenitor cells (HSPCs). After ex vivo activation and antigen loading, these autologous DCs could be re-administered into the cancer patient via various routes of administration (I.V., intravenous; I.N., intranodal; I.D., intradermal; and S.C., subcutaneous) to induce an antigen-specific T cell response against tumours [44]. The figure was created using www.app.biorender.com (accessed on 12 October 2023).

The application of vaccines in oncology has generated considerable excitement in recent years and represents a potential paradigm shift in how cancer could be treated and ultimately prevented. Three types of vaccine platforms or formulations have been developed for cancer therapy: cellular vaccines, vector-based vaccines, and molecular vaccines (DNA, RNA, peptides, and proteins) [37,38]. Despite the recent focus on molecular vaccines, the DC vaccine strategy (which falls under the cellular vaccine category) represents an exciting therapeutic avenue. With this strategy (shown in Figure 1), DCs’ superior antigen presentation machinery is exploited, whereby DCs are tumour-associated antigen (TAAs)-loaded and matured ex vivo before their autologous reinfusion into cancer patients in order to bypass TME immunosuppressive mechanisms and generate TAA-specific CTLs to initiate tumour cell killing and induce long-term immunological memory [2]. Although various clinical trials have confirmed the safety and feasibility of DC vaccines to induce an anti-tumour response, clinical patient responses remain poor [39]. Sporadic clinical responses are unsurprising considering the lack of a standardized protocol and the numerous variable factors involved in the design and execution of a DC vaccine strategy [40]. Such variable factors include the source and production of a DC cell type with potential inherent suboptimal antigen presentation and migration capacity. DC maturation stimuli, route of vaccine administration, frequency of injection, additional adjuvants, and overall competence of the patient’s immune system can also influence clinical outcomes. Initial DC vaccine strategies were designed to target advanced cancers, meaning the well-established active immunosuppression mechanisms of the TME provided a significant barrier to efficacy. The genomic instability of late-stage cancers results in heterogeneous tumour antigen expression, which again can hamper vaccines designed around a single epitope-targeting strategy. Despite these obstacles, the main technical issue with DC vaccines that needed to be overcome in order to make it a feasible translational avenue was the limited source material due to the low prevalence of DCs in the peripheral blood, ranging from 0.1–1% of peripheral blood mononuclear cells (PBMC) [41]. The original vaccine preparation attempts used density gradients to isolate an analogous APC-enriched preparation (leukapheresis) from a cancer patient’s own blood. This early strategy yielded Sipuleucel-T (Provenge^®^; Dendron Corporation, Seattle, WA, USA), a DC vaccine against asymptomatic or minimally symptomatic metastatic castration-resistant prostate cancer [42]. Sipuleucel-T was generated by exposing ex vivo cultured analogous APC-enriched preparations to a recombinant fusion protein consisting of the tumour antigen prostatic acid phosphatase (PAP) and granulocyte-macrophage colony-stimulating factor (GM-CSF). The IMPACT trial subsequently confirmed that Sipuleucel-T increased median overall survival (OS) rates by 3.9 months for castration-resistant prostate cancer patients [42]. Although modest, this clinical outcome was crucial in demonstrating the safety of the DC vaccine approach and led to subsequent FDA approval in 2010. Currently, further trials are underway with Sipuleucel-T in combination with other anticancer therapies in order to increase response rates [43].

Despite the FDA approval of Sipuleucel-T, the majority of early DC vaccine trials were disappointing. In a bid to improve response rates and low numbers of source materials, second-generation DC vaccine design initiatives centred on the ex vivo generation of MoDCs alongside the inclusion of maturation signals to produce a more reliable and readily available source of APCs that expressed high levels of co-stimulatory molecules. Monocytes (CD14^+^) compose ~10% of PBMC, and large numbers of MoDCs can be generated following treatment with GM-CSF and IL-4 [45,46]. Additional sources of DCs have stemmed from advancements in ex vivo DC derivation. CD34^+^ hematopoietic stem progenitor cells (HSPCs) from bone marrow and umbilical cord blood can now be used as a DC source material [47,48]. Indeed, HSPCs incubated in a cytokine cocktail of FLT3L, thrombopoietin (TO), and stem cell factor (SCF) can produce APCs with a primary DC phenotype distinct from MoDCs, capable of inducing more robust anti-tumour T cell responses [49,50]. Furthermore, the inclusion of maturation stimuli in any DC vaccine strategy is now one of general consensus, with the failure of early trials undeniably linked to the use of immature DCs that lacked sufficient expression of activation markers, with concomitant reduced migratory capacity and ability to stimulate T cell responses. Maturation strategies are constantly evolving to produce DCs that have a full activation status that can respond to secondary lymphoid organ chemokines and produce high levels of interleukin-12 (IL-12p70). Strategies can range from cytokine cocktails and TLR ligands to gene therapy. The Jonuleit cytokine cocktail (IL-1β, TNFα, IL-6, and prostaglandin E2 (PGE2)) was the original “gold standard” maturation strategy [2] until the substitution of PGE2 for IFNα (α type-1 polarised DC cocktail) was shown to produce DCs capable of producing higher levels of IL-12 and subsequently increase TAA-specific CTL numbers in a melanoma model [51,52]. The use of gene therapy to introduce endogenously expressed maturation signals (e.g., the TriMix platform) has resulted in the rapid induction of maturation without the additional requirement of cytokine cocktails. Such genetic modulation approaches could also contribute to remedying reliability and reproducibility issues in terms of ex vivo DC vaccine generation and maturation. In terms of future directions, next-generation DC vaccines will potentially involve the use of the cDC1 subset owing to their optimal ability to induce TAA-specific T cell responses [53,54,55]. However, robust strategies are still required to produce cDC1 cells ex vivo that resemble their primary counterparts at a scale that is amenable to therapeutic vaccine formulations.

Despite these evolving developmental steps, DC vaccines are showing continued benefits in the clinical setting, such as most recently in a phase III clinical trial in glioblastoma (GBM), the most lethal primary brain cancer (Clinical Trial ID: NCT00045968). The addition of autologous tumour lysate-loaded dendritic cell vaccine (DCVax-L) to standard of care temozolomide resulted in a statistically significant extension of survival for patients with both newly diagnosed and recurrent GBM [56]. Such results, especially in cancers with traditionally poor survival rates, have maintained the focus on DC vaccines as a therapeutic avenue. However, the main question now is “Have DC vaccines reached the maximum of their potential, or do they remain to be unlocked?”. The immunosuppressive mechanisms of the TME remain a major hurdle that hampers the efficacy of DC therapy and the ability of DC vaccines to mount robust anti-tumour responses. Hence, there is an urgent need for interventional strategies to boost and maximise the potential of DC vaccines. Currently, there are two main avenues of investigation. Firstly, bypass the immunosuppressive features of the TME by combining DC vaccines with FDA-approved treatment modalities that target these mechanisms (e.g., ipilimumab, tremelimumab) [40]. The second intervention strategy involves the genetic engineering of ex vivo-generated DCs using well-known molecular technologies (viral transduction, RNA interference (RNAi), and CRISPR/Cas9-mediated genome editing). Of note, the former strategy is beyond the scope of this review and is excellently reviewed elsewhere [40].

Our understanding of the biological roles of dendritic cells at the interface between the immune system and cancer enabled us to identify molecular targets for optimising their performance as a vaccination strategy. For instance, enhancing tumour-associated antigen (TAA) presentation [57] and lymph node migration [58], as well as enhancing immunogenicity [59,60], could be achieved by modulating the expression of different genes responsible for these anti-tumoural phenotypes. In this review, we discuss several attempts aimed at improving DC anti-tumoral functions and vaccine efficacy.

## 4. Genetic Engineering of DC-Based Vaccines to Improve Their Immunotherapeutic Potentials—A from within Approach

### 4.1. Viral-Based Approaches

Due to viruses’ natural capability to efficiently transduce eukaryotic cells with foreign nucleic acids, viral vectors have consistently been utilised as an efficient delivery vehicle for genetic modification purposes in academic and industrial laboratories for both research and clinical gene therapy applications. Viral vectors are classified according to whether viral infection results in transient short-term gene expression or permanent long-term gene expression following integration into the host genome. Additionally, depending on their genetic makeup, viruses can be classified into RNA-based and DNA-based vectors with either single-stranded (ss) or double-stranded (ds) genomes. Examples of the viral vectors include γ retroviruses, lentiviruses, adenoviruses, and adeno-associated viruses [61].

#### 4.1.1. CCR7

Manipulation of DCs’ ability to migrate to lymphoid tissues in order to prime CD8^+^ CTLs could dramatically improve DC vaccine efficacy. Numerous animal models have established that the chemokine receptor C-C chemokine receptor type 7 (CCR7), a G-protein-coupled receptor for the chemokine ligands CCL19 and CCL21, regulates DC chemotaxis, survival, and migratory speed in lymphoid tissue [62]. Indeed, high CCR7 expression levels in human tumours correlate with better clinical outcomes [63]. In 2005, Okada et al. proposed that genetically engineered DC vaccines to enhance CCR7 expression could produce a TAA-loaded DC that would efficiently home to a nearby lymphoid tissue and activate a robust T-cell response after administration—a strategy called “lymphoid tissue-directivity DC vaccine” [58]. Hence, a CCR7-overexpression bone marrow-derived DC vaccine was created by viral gene transduction using replication-deficient AdRGD (an adenovirus serotype 5 backbone with deletions of the E1 and E3 regions and the RGD sequence for AV-integrin targeting). Subsequent efficient AdRGD-mediated CCR7-gene transduction and overexpression into BMDCs were confirmed by semi-quantitative qRT-PCR analysis. Additionally, they reported that BMDCs—overexpressing CCR7 and cultured for 24 h—exhibited sufficient CCR7 protein localization on the cell surface in the flow cytometric analyses and showed strong migratory ability toward a CCL21 concentration gradient in a chemotaxis assay. Moreover, BMDCs expressing enhanced green fluorescent protein (eGFP) and transduced with AdRGD-CCR7 were demonstrated to migrate into the regional lymph nodes at approximately a 15-fold higher rate compared with mock DCs upon intradermal injection into the flank of wild-type mice.

#### 4.1.2. CD40L

CD40 Ligand (CD40L) or (CD154) is a type II transmembrane protein and a member of the tumour necrosis factor (TNF) superfamily of protein ligands [64]. Stimulation of the CD40 receptor on DCs by CD40L on activated CD4^+^ T lymphocytes (a process called DC licensing) results in the upregulation of co-stimulatory molecules (CD80/CD86) on DC surfaces, promotes proinflammatory cytokine production (e.g., IL-12, TNF-α, and interferon-γ (IFN-γ), facilitates the cross-presentation of antigens [64,65], and up-regulates CCR7 expression to enhance the capacity of DCs to migrate into secondary lymphoid tissues [66]. IL-12 is considered a Th1-polarising cytokine in both mouse and human DCs [4]. Additionally, it delivers a crucial cue for both Th1 T cell and CD8^+^ T cell differentiation and functions to induce potent anti-tumour cytotoxic T-cell immune responses. Ex vivo maturation of MoDCs by incubation with the Jonuleit cytokine cocktail showed some drawbacks [67,68]. For instance, matured MoDCs were found to be able to migrate but lack IL-12p70 expression [69]. To overcome this drawback, Ilka Knippertz and colleagues (2009) transduced MoDCs with an adenovirus vector coding for the trimeric human CD40L (Ad5hCD40L) in the presence of the Jonuleit cytokine cocktail in combination with a recombinant human IFN-γ treatment, which resulted in an increase in IL-12p70 expression and migration towards CCL19 [70].

### 4.2. RNA-Based Approaches

There are two main RNA approaches to enhance DC vaccine potential: firstly, mRNA transfection to overexpress genes involved in enhancing DC maturation; secondly, the use of RNA molecules, such as siRNAs, to knock down genes that suppress the immunotherapeutic potential of DCs.

#### 4.2.1. CD40L and TLR4

CD40L was introduced in the previous section and can increase DC expression of co-stimulatory molecules and ultimate maturation. Toll-like receptor 4 (TLR4) is a pattern recognition receptor that plays a central role in initiating innate immune cell responses and is involved in DC activation and pro-inflammatory cytokine production [71]. Hence, overexpressing TLR4 in DCs was determined to be a novel route to activate DCs without the need for cytokine cocktails. An example of a strategy to deliver mRNA in DCs was described by Calderhead et al. (2008) [72].CD40L mRNA was delivered by electroporation to cytokine-matured (treated with IL-1β, TNFα, IL-6, and PGE2) MoDCs, resulting in the long-term expansion of MART-1-reactive T cells that showed a CD28^+^/CD45RA^−^ effector/memory phenotype. In terms of TLR4 manipulation, Cisco et al. (2004) [73] showed that electroporation of a mRNA encoding a constitutively activated version of TLR4 (caTLR4) RNA into human DCs could lead to significant cytokine production and DC maturation (without the need for IL-12/TNF-γ-specific maturation) that led to enhanced allostimulation of CD4^+^ T-cells. Bonehill et al. (2008) combined both approaches through the delivery of mRNA species for CD40L, caTLR4, and CD70 into immature MoDCs as mRNA [74]. They demonstrated in vitro that the specific introduction of CD40L and caTLR4 into immature MoDCs could generate mature, cytokine-secreting DCs, mimicking CD40-CD40L and TLR4-LPS ligations. Furthermore, the co-introduction of CD70 provided a co-stimulatory signal to CD27^+^ naïve T-cells capable of blocking activated T-cell apoptosis and supporting T-cell proliferation. This combination was named TriMix and has been taken forward into preclinical trials where, instead of antigen pulsing, TriMix and MelanA-encoding (tumour-associated antigen) mRNA were electroporated together into DCs [35]. Results demonstrated the ability of this strategy to activate CD8^+^ T cells specific for tumour antigens. In a recent phase II clinical trial, TriMixDC-Mel in combination with ipilimumab was used to treat patients with pre-treated advanced melanoma [75]. In total, 39 patients were treated, and primary end-point data were collected at six months, where 38% of patients demonstrated anti-tumour responses. There were some common adverse events, such as skin reactions at the injection site (100%) and flu-like symptoms (84%); however, there were no grade 5 adverse events, and grade 3 or 4 immune-related adverse events were 36%. It was therefore concluded that this treatment was tolerable and showed tumour responses in the patient cohort tested. Fifteen patients were followed up for 5+ years, and their immune stimulation was measured [76]. After 390 weeks, 11 patients were alive, with 7 in complete remission. CD8^+^ T-cell responses for tumour-associated antigens were seen in all patients. Such findings clearly highlight the considerable potential for DC vaccines in vivo.

#### 4.2.2. IKKα and IKKβ

Ubiquitous NFκB transcription factors are central coordinators of immunity, inflammation, and cell survival [77]. In mammals, NFκB comprises a family of five proteins, forming ubiquitous dimeric complexes, NFκB1(p50)/RelA(p65) being the most abundant. In the canonical NFκB pathway, these dimers are normally held inactive, bound to IκB-family inhibitory proteins, and can be activated by signals causing the phosphorylation and proteolysis of IκBs by the IκBα-kinase (IKK) complex (typically comprising the two homologous catalytic subunits, IκB kinase α (IKKα) and IκB kinase β (IKKβ) and the regulatory scaffold subunit NEMO) and the proteasome, respectively [78]. NFκB thereafter enters the nucleus to activate transcription of a multitude of inflammatory mediators, immunoregulators, apoptosis inhibitors, and other genes, moulding the host defence responses to stress, injury, and infection [77]. NFκB pathway activation has been demonstrated to be crucial for DC maturation [79,80], and consequently, the overexpression of certain components of this pathway, in particular IKKα and IKKβ, could potentially open an additional avenue for mature DCs. Therefore, Pfeiffer et al. (2014) thought to activate this pathway through electroporation of constitutively active mutants of IκB kinases (caIKKα and caIKKβ) mRNAs in cytokine-matured human MoDCs [81]. DCs expressing these kinases had upregulated maturation markers, secreted higher cytokine levels (including IL-12), and induced CD27 expression in T cells in vitro. CD27 expression on T-cells indicates a memory phenotype demonstrating enhanced expansion capability, unlike traditional cytokine-maturated DCs alone. A follow-up study also demonstrated that these DCs can robustly activate autologous NK cells (which can directly lyse and kill tumour cells), as shown by the upregulation of CD54, CD69, and CD25 in vitro [82].

#### 4.2.3. PD-L1 and PD-L2

Programmed death 1 (PD-1) is involved in the control of immune tolerance and is one of the main protagonists in immune escape mechanisms during chronic viral infections and cancer [83,84]. The ligands of PD-1 (PD-L1 and PD-L2) are type I transmembrane glycoproteins that, upon binding to PD-1 on activated T and B cells, block T cell proliferation, cytokine production, and cell adhesion [83,84]. PD-L1 and PD-L2 expression have been demonstrated on the surface of APCS, including DCs. Consequently, siRNA silencing of PD-L1 and PD-L2 was undertaken by Hobo et al. (2010) in MoDCs [60]. Compared with the non-electroporated controls, these cells were shown to have the capacity to efficiently expand CD8^+^ effector and memory T cells in leukemic patients. Overall, DCs with PD-L1 and PD-L2 knockdown were demonstrated to enhance T cell proliferation and cytokine production. More recently, instead of electroporation, Hobo et al. showed that they could deliver the PD-L1 siRNA in lipid nanoparticles, resulting in the same DC phenotype as above [59].

#### 4.2.4. PTEN

Phosphatase and tensin homologue (PTEN) is a key negative regulator of the phosphatidylinositol 3-kinase (PI3K)/AKT pathway, which is involved in the activation of DCs [85,86]. Therefore, Kim et al. (2010) hypothesized that inhibition of PTEN could lead to heightened activation of DCs through unlocking the PI3K/AKT axis [87]. Indeed, down-regulation of PTEN in bone marrow-derived DCs (BMDCs) by transfection of PTEN siRNA was shown to result in AKT-dependent DC maturation [54]. Also observed was the upregulation of co-stimulatory molecules and the chemokine receptor CCR7, which demonstrated higher levels of in vitro T cell activation and generated higher numbers of tumour-specific CD8^+^ T cells in the HPV-16 E7-expressing murine tumour model [54].

### 4.3. CRISPR/Cas9-Based Engineering of DCs

**Figure 2:**
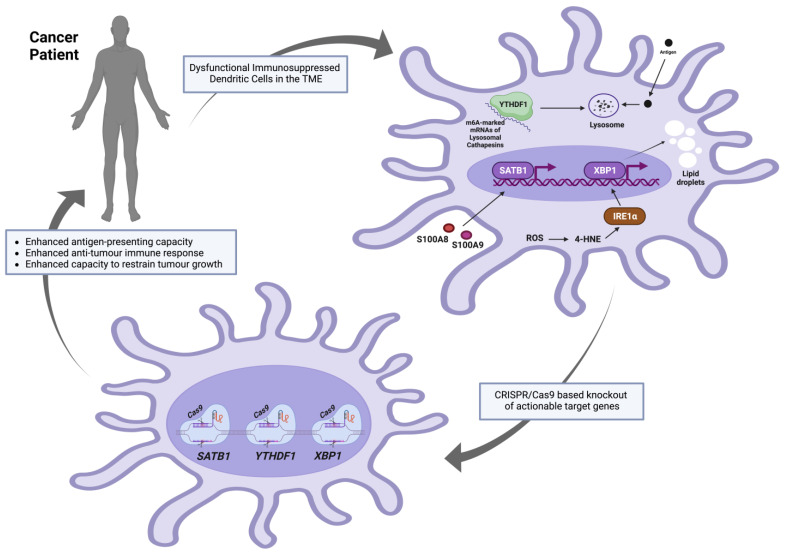
Proposed target genes to be knocked out in DCs using CRISPR/Cas9 approaches prior to autologous transfer to cancer patients. The figure was created using www.app.biorender.com (accessed on 12 October 2023).

The clustered regularly interspaced short palindromic repeats (CRISPR)/CRISPR-associated (Cas) system was discovered as a part of the adaptive immune system in bacteria that introduces site-specific double-stranded breaks (DSBs) in target foreign DNA by means of the dual RNA-guided DNA endonuclease Cas9 [88]. Since then, it has been widely used to modify targeted genes in eukaryotic cells and organisms and has emerged as a powerful tool for genome engineering. The induced DNA DSBs are usually repaired in eukaryotic systems by specific DNA-repair pathways (homology-directed repair [HDR], microhomology-mediated, end joining [MMEJ], and non-homologous end-joining (NHEJ]) [89]. Homology-directed repair (HDR) is a precise repair mechanism that depends on a homologous DNA template (a homologous sequence flanking the DNA cut site) to guide the repair of DSBs. MMEJ leads to deletions of DNA stretches of various lengths at the DNA break sites, resulting in the loss of sequence information. NHEJ leads to random insertions or deletions (InDels) of base pairs, resulting in frame shift mutations. Accordingly, MMEJ and NHEJ repair pathways functionally inactivate targeted genes with high efficiency [88]. The recent advancements of CRISPR/Cas9 gene-editing methodologies have made them more efficient (high specificity with minimal off-target effects), technically feasible, and promising to be included in DC-based vaccine manufacturing. If the actionable target genes that are implicated in the dysfunctional anti-tumoral roles of DCs do not severely affect the cell-cycling or cell viability phenotypes, such a strategy could be valuable in creating more potent genetically manipulated DC-based vaccines. Recent investigations, outlined below in Figure 2 and concluded in Table 1, revealed adverse molecular roles of several specific DC genes in the milieu of the tumour microenvironment (TME). It is worth noting that many other genes and signalling pathways have been suggested to be targeted to enhance the DC activation process. For instance, in the presence of TGF-β [90], DCs acquire a regulatory phenotype that favours the promotion of a tolerogenic immune response—a rationale to target TGFBR1 and TGFBR2 for genetic manipulation in DC-based vaccines. Additionally, interleukin-10 (IL-10) [91] produced by DCs efficiently inhibits proliferative and cytokine responses in T-cells, mediating immunological unresponsiveness and suppression of immune reactions—another rationale to ablate IL-10 in DC-based vaccines. However, the genes outlined below were reviewed based on favourable anti-tumoral phenotypes achieved by in vitro and/or in vivo knockdown or knockout in the context of tumorigenesis. Such experimental interventions have been demonstrated to be effective at enhancing anti-tumoral DC function in pre-clinical models.

#### 4.3.1. YTHDF1

YTH N6-methyladenosine RNA binding protein F1 (YTHDF1) is a member of a protein family—called “readers”—that can recognise the N6-methyladenosine (m6A) methylation as a post-transcriptional modification of mRNA transcripts. This family contains the YT521-B homology (YTH) domain; hence, the name YTHDF protein family [95]. In dendritic cells, YTHDF1 could bind to m6A-marked mRNAs encoding lysosomal cathepsins (proteases responsible for lysosomal-specific antigen degradation), promoting their translation and negatively affecting antigen presentation capabilities [57]. Recently, Dali Han and colleagues showed that the extent to which Ythdf1^−/−^ FLT3L-DCs (conventional DCs pulsed with necrotic B16-OVA cells in vitro) could cross-prime T cells expressing transgenic ovalbumin-specific (OT-I) T cell receptors was higher than the wildtype DCs. Additionally, at an in vivo level, CD8α^+^ and CD11b^+^ DCs from draining lymph nodes (DLNs) of B16-OVA- or MC38-OTIp-tumour-bearing Ythdf1^−/−^ mice showed a substantially augmented cross-priming capacity as compared with those collected from the wild mice when both co-cultured with OT-I T cells. Such an enhanced cross-priming capacity of CD8^+^ T-cells was attributed to the improved antigen presentation by DCs (as assessed by the abundance of H-2Kb-SIINFEKL complexes on DCs from wild-type and Ythdf1^−/−^ mice bearing B16-OVA tumours).

#### 4.3.2. XBP1

X-box binding protein 1 (XBP1) is a major transcription factor and a downstream effector of the inositol-requiring enzyme 1 (IRE1α) in the unfolded protein response (UPR)—a process that is usually initiated by endoplasmic reticulum (ER) membrane-bound sensors [96] to ensure protein folding fidelity and relieve the load of unfolded or misfolded proteins, restoring protein homeostasis (proteostasis) [97]. IRE1α-XBP1 activation and overexpression of various endoplasmic reticulum (ER) stress response markers were reported in ovarian cancer-associated DCs as compared with DCs isolated from non-tumorigenic normal tissues. This DC-specific ER stress was a result of the elevated levels of intracellular reactive oxygen species (ROS) that induced lipid peroxidation-generating reactive by-products (for example, the unsaturated aldehyde 4-hydroxy-trans-2-nonenal (4-HNE)) that could alter the ER-resident chaperone functions. Compounds that could sequester ROS or lipid peroxidation by-products (vitamin E or hydralazine, respectively) were reported to inhibit Xbp1 mRNA splicing and prevent the induction of the expression of ER stress response genes in ovarian cancer-associated DCs. Most importantly, the development and progression of p53/K-Ras-driven primary ovarian cancer were compromised in irradiated mice that were reconstituted with XBP1^f/f^ CD11c-Cre donor bone marrow as compared with control hosts transplanted with XBP1-sufficient (XBP1^f/f^) littermate bone marrow. Additionally, the same effects were observed in metastatic ovarian cancer models and were accompanied by the infiltration and accumulation of CD44^+^ IFNγ-secreting CD8^+^ and CD4^+^ T cells at tumour beds. Accordingly, the expression of XBP1 in CD11c^+^ DCs was reported to be critical for the initiation and progression of ovarian cancer, and its conditional ablation in DCs in the tumour microenvironment enhanced the capacity to restrict tumour growth. Finally, it was reported that aberrant XBP1 activation in tumour-associated dendritic cells led to lipid accumulation (due to aberrant triglyceride synthesis and accumulation), disrupting their normal antigen-presenting capacity, and XBP1-deficient ovarian cancer-associated DCs showed enhanced antigen-presenting capacity [33].

#### 4.3.3. SATB1

Special AT-rich sequence-binding protein-1 (SATB1) is a master coordinator of gene expression in various cell types. Through binding to the AT-rich motifs of the nuclear matrix attachment regions (MARS) of the DNA, it forms distinct loops, providing a “docking site” for chromatin remodelling proteins and transcription factors for the regulation of the expression of many genes. Such a SATB1-mediated nuclear organisation could control long-range regulation of genes located distal to the SATB1-bound loci. Accordingly, SATB1 integrates global epigenetic and transcriptional programmes, determining multiple cellular phenotypes [98]. Recently, Amelia J. Tesone and colleagues showed that although SATB1 could initiate a genome-wide transcriptional programme required for terminal steady-state DC differentiation and effective MHC II-mediated antigen presentation, its unremitting expression in fully committed inflammatory ovarian cancer-associated DCs drove an immunosuppressive phenotype, contributing to accelerated ovarian malignant progression. Additionally, SATB1 silencing in tumour-associated DCs boosted the anti-tumour immune response and delayed malignant progression [93]. Briefly, at an in vivo level, they found that small interfering RNA (siRNA)-loaded nanoplexes-based silencing of *Satb1*, specifically in tumour-associated dendritic cells of mice bearing ID8-Defb29/Vegf-a tumours, significantly enhanced anti-tumour immunity (assessed by the level of infiltration and accumulation of Granzyme B and IFN-γ-secreting antigen-experienced CD44^+^ cytotoxic T cells) as compared with non-targeting nanoplexes control. Additionally, upon in vivo pulsing Satb1-silenced tumour-associated DCs with the full-length ovalbumin (OVA), CD3^+^CFSE^+^OT-I^+^ T cells in situ responded to the cognate antigen in the ovarian cancer microenvironment stronger than those tumour-associated DCs in control mice treated with irrelevant nanoparticles. Most importantly, SATB1-specific silencing in tumour-associated DCs enhanced the survival rates of ID8-Defb29/Vegf-a-challenged mice [93].

## 5. Conclusions and Future Outlooks

Despite the steady evolution of DC vaccines, clinical responses to cancer remain sporadic. Such variability in response rates can be partly explained by the lack of consistency in manufacture when it comes to antigen loading, maturation protocols, source material, DC subtype, and trial design. Additionally, the immunosuppressive mechanisms that drive dysfunction in the TME can also potentially dampen the responses of even the most highly activated DC vaccines. Despite these obstacles, there is a pressing need to improve this branch of immunotherapy so that it can fully realise its potential. Although growing evidence points towards the cDC1 subtype as the ideal platform for next-generation DC vaccines due to its central role in tumour immunity and its superior ability to prime CD8^+^ T cells [99], a better understanding of DC biology and the immunosuppressive mechanisms that drive DC dysfunction remains essential if DC vaccine efficacy is to be augmented. In this review, we have identified several promising amenable targets along key immune modulatory pathways that could potentially unlock DC vaccines’ potential through the application of the latest genetic engineering tools. The revolution in the clinical application of genetic engineering, driven by CRISPR-based gene editing, means that for the first time, genetically edited DC therapies have a chance of future translation into the clinical setting. A point emphasised by the fact that CRISPR/Cas9 gene editing has revolutionised immunotherapy through the development of T-cell therapies, e.g., CAR-T-cell therapy, which have produced outstanding results in patients with hematologic malignancies and promising early results in solid tumours [100,101]. While the genetic manipulation of our highlighted targets is still at the in vitro and in vivo mouse model stage, the next step would be to move to clinical trials with these new approaches. The combination of modifications in terms of DC activation (e.g., CD40L and caTLR4), DC migration to lymph nodes (e.g., CCR7), and knockout of PDL1-driven immunosuppression could dramatically increase DC vaccine efficacy in cancer therapy. Of note, breakthroughs in genetic editing of DCs generated ex vivo could potentially lead to approaches to “re-educate” endogenous TME-resident DCs to a more tumoricidal phenotype. A concept that seems closer with the development of CRISPR-Cas9-Ribonucleoprotein and macrophage-targeted nano-assembly delivery systems to genetically edit our gene candidates in tumour-associated macrophages in vivo [102]. Such breakthroughs could rapidly expand the application of DC-based therapies beyond cancer to clinical initiatives to fight infectious diseases like HIV [103] or modulate DCs to promote the healing of chronic diabetes ulcers [104]. So, in a bid to answer our original question—How soon is now?—regarding the next generation of DC vaccines capable of generating robust anti-tumour responses: it is close, but important steps remain.

## Figures and Tables

**Table 1:** Proposed CRISPRR/Cas9-based gene ablations to improve the immunotherapeutic potential of DC-based vaccines.

Candidate Gene	Phenotype after Silencing/Knockout	Model System Investigated	References
YTHDF1	- Enhanced antigen-presentation capacity.	**In vitro** - Ythdf1^−/−^ FLT3L-DCs (conventional DCs pulsed with necrotic B16-OVA cells in vitro) co-cultured with OT-I naive CD8^+^ T cells. **In vivo** - CD11b^+^ or CD8^+^ conventional DCs collected from draining lymph node (DLNs) of B16-OVA- or MC38-OTIp-tumour-bearing Ythdf1^−/−^ mice and co-cultured with OT-I naive CD8^+^ T cells.	[57]
SATB1	- Enhanced anti-tumour immunity. - Enhanced antigen-presentation capacity. - Enhanced survival rates of tumour-challenged mice.	**In vivo** - (siRNA-loaded nanoplexes-based silencing of SATB1 specifically in tumour-associated DCs of mice bearing ID8-Defb29/Vegf-a tumours and pulsing Satb1-silenced tumour-associated DCs with the full-length ovalbumin (OVA)).	[92,93]
XBP1	- Enhanced anti-tumour immunity. - Enhanced antigen-presentation capacity. - Enhanced capacity to restrain tumour growth.	**In vitro** - Bone marrow-derived DCs (BMDCs) pulsed with full-length OVA protein in the presence of tumour-conditioned media (TCM) co-cultured with OT-1 T cells. - XBP1-deficient tumour-associated DCs (pulsed with full-length OVA or apoptotic OVA-expressing tumour cells in the presence of ascites) co-cultured with OT-1 T cells. **In vivo** - p53/K-Ras-driven primary and metastatic ovarian cancer in irradiated mice reconstituted with XBP1^f/f^ CD11c-Cre donor bone marrow	[92,94]

## Data Availability

No new data were created or analyzed in this study. Data sharing is not applicable to this article.
